# Supplementary material for: A Mid-Atlantic Brook Trout (Salvelinus fontinalis) genome to advance conservation and comparative genomics
Source: G3 (Bethesda). 2026 Aug 2;16(10):jkag209. doi: 10.1093/g3journal/jkag209 (PMC13643803; doi:10.1093/g3journal/jkag209)
Supplement: Supplementary file 1 [file jkag209_supplementary_data.docx]

## Title page

**Title:** A Mid-Atlantic Brook Trout (*Salvelinus fontinalis*) genome to advance conservation and comparative genomics

**Running Title:** Mid-Atlantic Brook Trout genome

**Authors:** Samuel W. Rosenbaum^1*^, Merly Escalona^2,3^, Samuel A. May^4^, Andrew R. Whiteley^1^

**Affiliations:**

1. Wildlife Biology Program, Department of Ecosystem and Conservation Sciences, Franke College of Forestry and Conservation, University of Montana, Missoula, MT, USA
2. Institute for Systems Genomics, University of Connecticut, Storrs, CT, USA
3. Department of Biomolecular Engineering, University of California, Santa Cruz, Santa Cruz, CA, USA
4. Center for Aquaculture Technologies, San Diego, CA, USA

* Corresponding author: [sam.rosenbaum@umontana.edu](mailto:sam.rosenbaum@umontana.edu), <https://orcid.org/0009-0004-5197-5841>

**Keywords:** Mid-Atlantic Brook Trout, *Salvelinus fontinalis*, Genome Assembly, Reference Bias

## Abstract

Brook Trout (*Salvelinus fontinalis*) are experiencing genomic erosion and demographic declines across the southern portion of their native distribution. A regionally representative reference genome is necessary to support conservation genomic initiatives for this species. While Brook Trout exhibit substantial phylogenetic structure across their range, only a single reference genome from Northeastern Canada (ASM2944872v1) is currently used to guide range-wide analyses. Consequently, reference bias is expected to cause erroneous sequence alignment and spurious variant detection for Brook Trout from divergent lineages. To prevent reference bias and invalid inference when studying Mid-Atlantic Brook Trout, we assembled a *de novo*, chromosome-level genome using a wild individual from Virginia, USA and produced a gene annotation with publicly available RNA-seq data. The assembly combined three complementary sequencing types (PacBio HiFi, Oxford Nanopore Ultra-Long, and Dovetail Omni-C) and benefited from manual curation using the Pretext software suite. The 2.91 Gb genome, known as mSalvFont1.0, consists of 3,333 contigs (N50 = 3.3 Mb) and 1,299 scaffolds (N50 = 55 Mb), with 81% of the assembly contained within 42 chromosome-scale scaffolds. Indices of accuracy and completeness reveal a quality value of 59 (99.999% accuracy), k-mer completeness of 95%, and 99.3% complete BUSCOs from the Actinopterygii orthologous gene set. Additionally, we identified reference bias (26.94% heterozygosity inflation) when variant detection of a Mid-Atlantic-origin sample relied on alignment to ASM2944872v1, instead of mSalvFont1.0. We anticipate that mSalvFont1.0 will increase the accuracy and precision of regional conservation genomic initiatives and expedite the genesis of a Brook Trout pangenome.

## Introduction

Innovations in computing and DNA sequencing technologies are accelerating the integration of genomic methods within conservation frameworks (May et al. 2025), with most conservation genetic analyses depending on linear reference genomes to align novel sequencing data (Formenti et al. 2022). However, assembly errors and phylogenetic divergence among references and samples may introduce reference bias, where differentiated reads fail to map or become misaligned and drive incorrect variant calls. Such reference bias can reduce data quantity and quality, and ultimately obscure inference (Bohling et al. 2020; Prasad et al. 2022; Rick et al. 2024; Akopyan et al. 2025; Mohn et al. 2026). This systematic loss of valid information is particularly prevalent in studies of non-model species, which often rely on a singular reference genome to inform range-wide exploration (Brandies et al. 2019; Paez et al. 2022). As genome assembly resources become more affordable and accessible (Li & Durbin 2024), the generation of local references will mitigate reference bias, improve regional discovery, and enable broader comparative genomic pursuits (Thorburn et al. 2023).

An excellent example of a species warranting regional genome assemblies is the Brook Trout (*Salvelinus fontinalis*), a prominent salmonid with a natural distribution spanning latitudinally from Northeastern Canada to the Southeastern United States, and longitudinally from the Laurentian Great Lakes to the Atlantic Ocean (Hudy et al. 2008; Whiteley et al. 2019). Contemporary Brook Trout structure has been attributed to allopatric divergence driven by historical glacial isolation (Danzmann et al. 1998). While whole-genome sequencing (WGS) approaches remain underutilized for researching Brook Trout in the United States (but see Canadian WGS efforts: Brookes et al. 2022; Beemelmanns et al. 2025; Nemeczek et al. 2025), previous population genetic studies (e.g., using microsatellites or RAD-seq) have uncovered support for a minimum of three genetic clusters (Mississippian, Mid-Atlantic, and Acadian; Kazyak et al. 2022) and possibly five (Mamoozadeh et al. 2023) that could represent historical neutral and adaptive genetic divergence consistent with Evolutionarily Significant Units (ESUs; Waples 1991; Funk et al. 2012).

Although precise, fine-scale patterns of divergence among biogeographic groups of Brook Trout remain largely unexplored with WGS (but see Meek et al. 2025 for RAD-seq estimates of private genetic variation), it is likely that each lineage evolved refugia-specific adaptations during glacial isolation. Brook Trout exhibit a diverse array of heritable behavioral, morphological, and physiological traits across their range (Hutchings 1996) and occur in a highly fragmented (Hudy et al. 2008) and now anthropogenically influenced landscape (Merriam et al. 2019), leading to a strong potential for contemporary genomic divergence that results from both neutral and adaptive processes (Erdman et al. 2024; Jeon et al. 2024; Mamoozadeh et al. 2026). Further, substantial structural variation is present within and across several salmonid species (Bertolotti et al. 2020; Liu et al. 2021; Tigano & Russello 2022; Mérot et al. 2023; Tigano et al. 2023; Lecomte et al. 2024; Ali et al. 2026) and indirect evidence has suggested putative inversions occur in some Canadian Brook Trout populations as well (Nemeczek et al. 2025). Developing phylogenetically distinct Brook Trout reference genomes will improve the detection of overlooked structural and nucleotide variation, increase the resolution of eco-evolutionary studies among lineages, and provide the foundation for a comprehensive pangenome.

Pervasive population declines, coupled with ecological and economic significance, necessitate genetic monitoring programs to inform Brook Trout conservation in the United States and Canada (Jones et al. 1996; White et al. 2023; Robinson et al. 2024; Schwinghamer et al. 2024). To date, only a single genome assembly, ASM2944872v1 (Lecomte et al. 2025), has served as the sole Brook Trout reference for such efforts. This assembly was generated from a double-haploid female (i.e., entirely homozygous) from the northernmost extent of the species range in Newfoundland, Canada (Figure 1). While this assembly can accurately align sequences from specimens within close phylogenetic distances to what we hereafter refer to as the Northern Brook Trout, following Mamoozadeh et al. (2023), reference bias may obscure inference when studying individuals from divergent biogeographic regions (Thorburn et al. 2023).

In this study we assembled a *de novo*, chromosome-level Brook Trout genome from Virginia, USA, using recently developed bioinformatic and sequencing techniques. Known as mSalvFont1.0, this Mid-Atlantic Brook Trout genome will improve accuracy and precision in conservation genomics across the more southern portion of the species’ range. mSalvFont1.0 will additionally enable broader comparative analyses within and beyond *Salvelinus*, delivering a promising tool for expanding genome-wide exploration of salmonid diversity. Looking ahead, we envision that mSalvFont1.0 will serve as a foundational building block for the future construction of a Brook Trout pangenome.

## Methods

### Sampling and Sequencing

A wild male Brook Trout was captured alive using a backpack electrofishing unit on November 7, 2023, from Dry Run, Virginia, USA (Figure 1). This isolated tributary is located within the North River watershed of the Potomac River basin and is part of the George Washington National Forest (Robinson et al. 2017). The specimen was humanely euthanized following an IACUC-approved protocol (AUP 057-23AWWB-102723). The heart, dorsal fin, and testes were promptly dissected and immediately flash-frozen with liquid nitrogen in the field. Heart and dorsal fin samples were transported to the University of Maryland Genomics Core for PacBio HiFi sequencing, while testes were shipped on dry ice to Dovetail Genomics for Omni-C sequencing. Later, the remaining frozen tissue and extracted high-molecular-weight (HMW) DNA from the heart and dorsal fin samples were shipped to Novogene Corporation, Inc., for Oxford Nanopore Technologies (ONT) Ultra-Long (UL) Simplex sequencing. Additionally, at the time of euthanasia, pectoral fin tissue was dissected and preserved on Whatman paper for short-read WGS to facilitate downstream reference bias quantification.

HMW DNA was extracted from approximately 25 milligrams of flash-frozen heart tissue at the University of Maryland Genomics Core using the Nanobind Tissue kit (Circulomics, Baltimore, MD, USA). HiFi libraries were prepared by size-selecting DNA fragments between 15 and 20 Kilobases (Kb) and ligating them with PacBio adapters. To generate a minimum target of 30x coverage per haplotype, two PacBio Revio SMRT Cells were sequenced in HiFi mode (24-hour movie sequencing runs). The remaining flash-frozen heart and dorsal fin samples were sent to Novogene Corporation, Inc. (Davis, California) for HMW extraction using the PacBio PanDNA kit (catalog 103-260-000) for UL library preparation. HMW DNA was then shipped to Novogene’s overseas facility in China, where a Simplex library was constructed using the ONT Ligation Sequencing Kit V14 (SQK-LSK114) and sequenced on a single R10.4 ONT PromethION flow cell, targeting UL reads of length ≥ 50 Kb. Frozen testes tissue was shipped on dry ice to Dovetail Genomics (Scotts Valley, California) for library construction using the proprietary Dovetail Omni-C Kit, following the manufacturer’s protocol. The Omni-C library was generated using a sequence-independent endonuclease-based process, which enabled unbiased, increased genome-wide coverage (<https://cantatabio.com/dovetail-genomics/product-literature/>). Chromatin was crosslinked within the nucleus, digested, ends repaired, ligated with biotin, and then prepared for paired-end Illumina short-read sequencing on a single NovaSeq X Plus lane.

Lastly, genomic DNA was extracted from pectoral fin tissue at the Montana Fish and Wildlife Genomics Lab following a modified version of the protocol detailed in Ali et al. (2016). Specific deviations included 1) the use of Sera-Mag carboxylate-modified magnetic particles (Cytiva, Marlborough, MA, USA) instead of AMPure XP beads, and 2) overnight sample incubation at 37° Celsius. Concentration of genomic DNA was quantified with the PicoGreen dsDNA Reagent (Invitrogen, Carlsbad, CA, USA). A WGS short-read library was constructed with the NEBNext Ultra II FS DNA Library Preparation Kit for Illumina (New England Biolabs, Ipswich, MA, USA; catalog E7805), using an input of 300 nanograms of genomic DNA and performing three minutes of fragmentation. Next, size selection for DNA fragments between 300–500 bp was performed with AMPure XP beads (Beckman Coulter, Brea, CA, USA; catalog A63881) and the resulting library was enriched with six cycles of PCR amplification using NEBNext Unique Dual Indexes (New England Biolabs). The final genomic library was sent to Novogene Corporation, Inc. (Davis, California) for sequencing on a NovaSeq X Plus instrument using a 25B paired-end 150 bp (PE150) read configuration.

### Assembly

Table S1 contains information on software versions and non-default parameters used in the analyses described below. First, summary statistics were calculated from the raw HiFi and UL reads with NanoStat (De Coster et al. 2018). HiFi sequences containing adapter contamination were removed using HiFiAdapterFilt (Sim et al. 2022), and UL reads were adapter-trimmed with Dorado (<https://github.com/nanoporetech/dorado>). Statistics from trimmed reads were then recalculated. Preliminary assembly parameters based on k-mer counts were estimated from adapter-filtered HiFi reads. K-mers of size 21 were identified from the read set using meryl (Rhie et al. 2020). Genome length, repetitive content, coverage, and heterozygosity were then estimated using GenomeScope2.0 (Ranallo-Benavidez et al. 2020).

Adapter-trimmed HiFi and UL reads (i.e., ≥ 50 Kb), in combination with Omni-C data, were used to assemble a haploid representation of the Brook Trout genome with hifiasm (Cheng et al. 2024). Falsely duplicated haplotypes were removed using hifiasm’s internal purging tool with a similarity threshold set to 0.55. Removal of remaining false duplications occurred during manual curation (see below). Hifiasm produced contigs in GFA format, which were then converted to FASTA format using gfatools (<https://github.com/lh3/gfatools>).

Omni-C reads were aligned to contigs using BWA-MEM (Li 2013), following the manufacturer’s recommended bioinformatic pipeline (<https://omni-c.readthedocs.io/en/latest/index.html>). To identify and order ligation events, remove PCR duplicates, record ligation and sequencing statistics, and create a pairs file, pairtools was used (Open2C et al. 2024). Next, a sorted and indexed BAM file was generated using samtools (Li et al. 2009). Aligned reads were subsequently used to categorize Hi-C links with pairtools by distance and class. An initial exploratory Hi-C contact map of genome-wide chromatin interactions was generated with filtered Omni-C data using PretextMap and PretextSnapshot (<https://github.com/sanger-tol>).

Contigs were organized into scaffolds using the FASTA file and the Omni-C pairs file with YaHS (Zhou et al. 2023). Default parameters were used, with the exception of disabling contig and scaffold error-correction flags because manual curation (e.g., breaking, joining, flipping, or discarding sequences based on chromatin interactions, HiFi coverage, and gaps) serves as a conservative and complementary step to aid the generalized heuristics encoded within automated software (Howe et al. 2021). Omni-C reads were re-aligned to the scaffold-level assembly following the steps described above, and a scaffold-level Omni-C map was generated using the Pretext software suite (<https://github.com/sanger-tol>). Omni-C alignment statistics (e.g., total read pairs, mapped read pairs, PCR duplicated read pairs, and non-duplicated valid read pairs) were produced with Dovetail’s get_qc.py script (<https://github.com/dovetail-genomics/Omni-C/blob/main/get_qc.py>). Next, manual curation of the scaffolded assembly was conducted following the Wellcome Trust Sanger Institute’s rapid curation pipeline (<https://github.com/sanger-tol/curationpretext>), which included visual inspection and manipulation of scaffolds based on continuity of chromatin contact frequencies, gap locations, and HiFi coverage using PretextView (<https://github.com/sanger-tol/PretextView>). Overall, curation efforts aimed to optimize the contact map’s accuracy by increasing chromatin self-affinity matches through resolution of off-diagonal associations. Guides provided by <https://github.com/sanger-tol/curation-resources> were followed to assist with diagnosis and remediation of imperfections introduced by automated tools (e.g., hifiasm and YaHS).

Following manual curation, a fraction of the remaining gaps in the assembly were then closed with YAGCloser (<https://github.com/merlyescalona/yagcloser>), which conservatively filled gaps using the filtered HiFi reads. A curated Omni-C contact map was subset to only include the 42 largest chromosome-scale scaffolds, matching the expected Brook Trout karyotype (Lee et al. 1981; Śliwińska-Jewsiewicka et al. 2015). To construct and annotate the circular mitochondrial genome, this study’s unassembled contigs and a previously deposited Brook Trout mitochondrial sequence (NCBI Accession: NC_000860) were input to the MitoHifi software for a reference-guided assembly (Uliano‐Silva et al. 2023). Refer to Figure 2 for a conceptual diagram of the major assembly steps described above.

Both Mid-Atlantic (mSalvFont1.0) and Northern (ASM2944872v1) Brook Trout genomes were subset to their 42 chromosome-level scaffolds (i.e., unallocated scaffolds were discarded) and aligned to one another using the mum algorithm in mummer4 (Marcais et al. 2018). Note that the full version of mSalvFont1.0, as available on NCBI (GCA_053542365.1), includes both chromosome-scale and unallocated scaffolds. To aid visual comparisons of large synteny blocks, macrosyntenic regions between Brook Trout assemblies were identified with ntSynt and visualized with ntSynt-viz (Coombe et al. 2025; Coombe et al. 2025). A dot plot comparing chromosome-scaffolds across assemblies was then generated using dotPlotly (<https://github.com/tpoorten/dotPlotly>), after reformatting the mummer4 DELTA file. To facilitate comparisons among Brook Trout genomes, chromosomes were ordered and named in mSalvFont1.0 based on their synteny, but not orientation, with ASM2944872v1. Additionally, to produce a repeat-masked version of mSalvFont1.0’s 42 chromosome-scale scaffolds and obtain a canonical estimate of repetitive content, RepeatModeler (Flynn et al. 2020) was used to discover and annotate interspersed repeats and low-complexity DNA throughout Mid-Atlantic Brook Trout chromosomes, and then RepeatMasker was used to hard-mask these sequences (Smit et al. 2015).

### Quality Assessment

Assembly statistics were calculated using Gfastats (Formenti et al. 2022). Benchmarking Universal Single-Copy Orthologs (BUSCOs) were checked against the assembly to assess gene completeness using the Actinopterygii odb10 dataset (Simão et al. 2015; Manni et al. 2021). Additionally, per-base quality value (QV) and k-mer completeness metrics were quantified using Merqury by comparing the k-mer database generated from the HiFi reads with mSalvFont1.0 (Rhie et al. 2020). To screen the assembly for contamination from non-target DNA, a custom Kraken2 database was built (Wood et al. 2019) that included archaea, bacteria, fungi, human, protozoa, and viral genomes, as well as the Northern Brook Trout genome (ASM2944872v1). For further validation, the assembly was screened with NCBI’s Foreign Contamination Screening FCS-GX pipeline (Astashyn et al. 2024). Inspection of non-target DNA with the tools listed above occurred both before and after manual curation, while removal of putative contamination occurred only following completion of curation efforts.

### Annotation

A *de novo* gene annotation was produced using mSalvFont1.0’s 42 chromosome-scale scaffolds and a publicly accessible repository of Brook Trout RNA-seq data (Pasquier et al. 2016), with the Eukaryotic Genome Annotation Pipeline - External (EGAPx) (<https://github.com/ncbi/egapx>). This gene expression database contained transcriptomic paired-end reads from 11 different Brook Trout tissue types, including embryo, testis, intestine, bone, kidney, liver, muscle, heart, gill, brain, and ovary (NCBI: PRJNA256998). EGAPx is NCBI’s publicly available version of their widely utilized Eukaryotic Genome Annotation Pipeline, which leverages a combination of *ab initio* and homology modeling to build robust gene predictions (<https://www.ncbi.nlm.nih.gov/refseq/annotation_euk/process/>).

### sdY Verification

The salmonid master sex-determining gene, sdY (sexually dimorphic on the Y chromosome), appears to be conserved within Brook Trout (Yanno et al. 2013; Hale et al. 2017; Webster et al. 2021). Sequencing of a Brook Trout sdY transcript identified an mRNA partial coding sequence (CDS; 515 bp) that is available through NCBI (GenBank: JF826021). mSalvFont1.0 is a primary assembly (i.e., an unphased, haploid representation of the genome), which reduced the likelihood of capturing the single-copy CDS in this haploid mosaic of paternal and maternal sequences. To determine whether the sdY CDS was present within any chromosome-scaffolds, the reference sequence (JF826021) was queried against the 42 Brook Trout chromosomes using BLASTN (Altschul et al. 1997; McGinnis et al. 2004). Given evidence that female salmonids can carry multiple autosomal copies of non-functional sdY pseudogenes (Bertho et al. 2022), chromosome-level BLASTN results were validated by screening the female ASM2944872v1 assembly, which should not contain a functional sdY. As final validation steps, both the HiFi reads used for initial contig generation and the full Mid-Atlantic genome (mSalvFont1.0), containing unplaced scaffolds and chromosome-scale scaffolds alike, were queried for the sdY CDS.

### Salmonid Comparisons

To benchmark the accuracy and contiguity of the curated Mid-Atlantic Brook Trout assembly, mSalvFont1.0 was compared with three other North American trout genomes published in 2025. Specifically, the *de novo* Mid-Atlantic Brook Trout genome (*Salvelinus fontinalis*; GCA_053542365.1; mSalvFont1.0) was measured against the Northern Brook Trout (*Salvelinus fontinalis*; GCA_029448725.1; ASM2944872v1; Lecomte et al. 2025), Lahontan Cutthroat Trout (*Oncorhynchus henshawi*; GCA_043774065.1; Payne et al. 2025), and Westslope Cutthroat Trout (*Oncorhynchus lewisi*; GCA_045791955.2; Flores et al. 2025) assemblies. Metrics regarding assembly contiguity (i.e., total number of contigs, scaffolds, and gaps) and accuracy (i.e., complete BUSCOs, quality value, and k-mer completeness) were retrieved from the relevant publications.

### Inversion Validation

A chromatin contact map for the Northern Brook Trout (ASM2944872v1) was generated with the same pipeline applied to mSalvFont1.0 following Hi-C data retrieval from NCBI (SAMN32676627; Lecomte et al. 2025), which enabled nuanced scrutiny of synteny and karyotypic congruence among Brook Trout genomes. To assess whether apparent structural differences among genomes were the result of true biological signal versus false assembly noise, an additional two contact maps were generated using the aforementioned bioinformatic pipeline by aligning 1) the Northern Hi-C data to the Mid-Atlantic assembly and 2) the Mid-Atlantic Omni-C data to the Northern assembly. This cross-mapping exercise enabled deterministic validation of putative lineage-specific inversions flagged during inspection of macrosyntenic blocks.

### Reference Bias

A major outstanding question in the resource-limited field of conservation genomics concerns the effectiveness of single, linear reference genomes to accurately anchor sequences and reliably identify polymorphisms for broadly distributed or diverging species without severe analytical repercussions. To quantify the extent to which reference bias occurs when the Northern Brook Trout genome (ASM2944872v1) is used to align reads and call variants for a Mid-Atlantic Brook Trout sample, WGS reads were generated from pectoral fin tissue collected from the same specimen used to assemble mSalvFont1.0. First, to reduce noise from unallocated sequences, both Brook Trout assemblies were subset to only include their 42 chromosome-scale scaffolds. Then, the same set of Mid-Atlantic Brook Trout reads were mapped to each assembly and variants were identified using an otherwise identical execution of the snpArcher pipeline (Mirchandani et al. 2024). This semi-automated workflow is modern, reproducible, and optimized for efficiently processing non-model WGS samples by mapping reads with the Burrows-Wheeler alignment algorithm (Li 2013) and identifying variants with GATK (McKenna et al. 2010). To establish how reference genome choice affects mapping success, the percent of mapped, duplicated, and properly paired reads, plus mean depth, were collected from snpArcher’s quality control module for each instance of the pipeline. To determine differences in raw variant identification, BCFtools (Danecek et al. 2021) was used on both resulting VCF files to calculate raw numbers of SNPs, INDELs, multiallelic sites, and heterozygous sites. Next, to assess how variant filtering mitigates reference bias, stringent criteria including flagging of low-quality sites (< 99.9% variant call accuracy), low read depth (< 10x), excessive read depth (> 100x), and low-quality genotypes (< 99% confidence) were applied and variant statistics were recalculated.

## Results and discussion

### Sequencing

Integration of high-accuracy HiFi data, UL reads to disentangle complex and repetitive regions, and unbiased Omni-C sequences enabled the generation of a robust primary genome assembly for Mid-Atlantic Brook Trout (mSalvFont1.0). A total of 8.48 million HiFi reads (total yield 157 giga base pairs (Gb); mean read length = 18.5 Kb; mean read quality score = Q23), 0.323 million UL reads (total yield 16.2 Gb; mean read length = 50.1 Kb; mean read quality score = Q16), and 423 million Omni-C read pairs were generated. Comprehensive metrics for HiFi and UL reads were reported in Table S2, while Omni-C alignment statistics were presented in Tables S3 and S4. Additionally, Illumina WGS produced 808 million read pairs using tissue from the same individual used to assemble mSalvFont1.0 for reference bias analyses.

### Parameter Estimation

A haploid genome size of 2.18 Gb, with 61.6% repetitive content (38.4% unique, single-copy k-mers), 99.2% homozygosity, 35x coverage of heterozygous sites, and a sequencing error rate of 0.131% were estimated using k-mers from the HiFi reads (Figure 2, bottom left inset). The estimated genome length was 0.73 Gb smaller than the final curated assembly size (mSalvFont1.0). Factors contributing to discrepancies between estimated and assembled length may include the substantial repetitive content (~61.6%) and the pseudo-tetraploid state of salmonid genomes following ancient whole-genome duplication (WGD) (Allendorf et al. 2015). The genome size estimation was conducted using the k-mer frequency spectrum (Ranallo-Benavidez et al. 2020), and substantial or improperly modeled sequence duplication and repetitiveness may have skewed the results, a concern especially relevant for salmonids undergoing rediploidization. Underestimation of the true assembled size of the Lahontan Cutthroat Trout genome (0.34 Gb difference) (Payne et al. 2025) provides additional evidence that GenomeScope2.0 may systematically underestimate salmonid genomes, as well as those of other taxa (Pflug et al. 2020).

### Assembly

The initial draft genome was 3.00 Gb and consisted of 3,369 contigs (contig N50 = 3.3 Mb) following *de novo* contig assembly (Table 1). Next, 1,391 scaffolds (scaffold N50 = 68 Mb) were joined by connecting contigs into larger, gapped sequences, and later, 200 breaks, 383 joins, and removal of 116 falsely retained haplotypic duplications were made during manual curation. We assert that manual curation continues to be a crucial yet underused step in the assembly process (Howe et al. 2021; Rhie et al. 2021) and has the potential to yield outsized benefits for the assembly of highly complex salmonid genomes (following <https://curation.tol.sanger.ac.uk/>).

An additional 61 gaps were closed based on spanning of HiFi reads, the circular mitochondrial genome was assembled, and potentially contaminated, unallocated scaffolds were removed following manual curation. The archived Brook Trout mitochondrial sequence (NC_000860) used for organelle assembly contained 16,624 base pairs and 37 genes. mSalvFont1.0’s assembled circular mitochondrial genome includes 16,623 base pairs and 37 genes. Contamination screening before and after manual curation revealed 99.57% of scaffolds were classified as *Salvelinus fontinalis*, while 0.43% were unclassified. These nine unclassified scaffolds were removed following curation because they remained unallocated (i.e., not assigned to a chromosome-scaffold) after modifying the chromatin contact map. The final assembly (mSalvFont1.0) was reduced to 2.91 Gb and is comprised of 3,333 contigs (contig N50 = 3.3 Mb) and 1,299 scaffolds (scaffold N50 = 55 Mb) after manual removal of false haplotypic duplications. Forty-two chromosome-level scaffolds were identified during curation, which is consistent with Brook Trout karyotyping efforts (Lee et al. 1981; Śliwińska-Jewsiewicka et al. 2015). Assembly size was further reduced to 2.36 Gb when subset for visualization purposes to only include the 42 chromosome-scaffolds (‘Chromosome-Scale’, Table 1). More than half of the bases (55.23%; 1.30 Gb) contained within these 42 chromosome-scale sequences (i.e., excluding unallocated scaffolds) were identified as repetitive content, which differs from the 61.6% estimated by k-mer analysis conducted on the entire unassembled HiFi read set. The elements identified and summarized by RepeatModeler and RepeatMasker can be broadly classified within chromosome-scaffolds as 51.99% interspersed repeats, 2.51% simple repeats or low complexity regions, and 0.74% small RNAs. A complete characterization of all repetitive sequences can be found in Table S5, and the resulting repeat-masked, chromosome-subset genome is publicly available (<https://doi.org/10.5281/zenodo.21083596>).

### Quality Assessment

The curated assembly (mSalvFont1.0) contains 99.3% complete BUSCOs found in Actinopterygii odb10, while the chromosome-scale subset includes 94.7% complete BUSCOs (Table 1). mSalvFont1.0 retains 95% of the k-mers present within all HiFi reads, indicating thorough assembly completeness. The accuracy of mSalvFont1.0 was substantial, with a Phred-scored quality value (QV) of 59, equivalent to a single error per every 794 Kb (i.e., 99.999% accuracy). These metrics suggest that the final curated assembly is both highly complete and accurate, while the chromosome-scale subset is accurate but likely misses up to 4.6% of the true gene content.

### Annotation

The EGAPx annotation identified 43,790 genes, 69,297 mRNAs, 3,302 non-coding RNAs, and 5,281 pseudo transcripts contained within mSalvFont1.0’s 42 chromosome-scale scaffolds. A partial accounting of annotation features is presented in Table 2, while more comprehensive metrics are located within Table S6. The annotation and accompanying outputs from EGAPx are available for public access (<https://doi.org/10.5281/zenodo.21083596>). This resource will enable deeper insights into lineage-specific gene family expansions and contractions unique to Mid-Atlantic Brook Trout.

### sdY Verification

A complete match for the Brook Trout sdY mRNA partial CDS was not identified within the 42 chromosome-scaffolds in either Mid-Atlantic (mSalvFont1.0; male) or Northern (ASM2944872v1; female) Brook Trout assemblies. However, three partial hits were identified in both chromosome-subset assemblies, each showing congruent alignment lengths, percent identities, and E-values. Such parallel results between male (Table 3, Rows 1-3) and female (Table 3, Rows 5-7) Brook Trout genomes suggest that these hits identified partially diverged non-functional autosomal sdY duplicates (pseudogenes) that arose during WGD (Bertho et al. 2022; Cisneros et al. 2026). Considering that ASM2944872v1 was assembled with a female sample, it is expected to lack an intact sdY sequence while still retaining pseudogene copies (Payne et al. 2025). Of note, these partial hits were not located on syntenic chromosomes among assemblies. The functional sdY gene is thought to translocate among chromosomes within salmonid species (Faber-Hammond et al. 2015; McKinney et al. 2021), and these results potentially provide evidence that sdY pseudogenes may behave similarly. Several studies have reported phenotypic and genetic discordance in salmonid sex identification with sdY assays, thought to be driven by the complicating presence of such pseudogenes (Ayllon et al. 2020). Recent salmonid assemblies from male specimens, or of those of unknown sex, have also failed to place sdY among chromosome-scale scaffolds (Rondeau et al. 2023; Christensen et al. 2025; Payne et al. 2025; Flores et al. 2025; Hudgell et al. 2025), likely owing to this gene’s small size and surrounding repetitive content (Lubieniecki et al. 2015).

Screening of the raw mSalvFont1.0 HiFi reads for the sdY CDS was performed to confirm its presence in the unassembled data, revealing 466 statistically significant hits across the read set (i.e., E-value < 1e-5; full data available <https://doi.org/10.5281/zenodo.21083596>). Reads displaying the most statistical support (i.e., greatest alignment lengths, highest percent identity, and lowest E-values) revealed a consistent pattern of three partial (< 370 bp), high-identity (> 99.0%), low E-value (< 3e-11) hits per flagged read (Table 3, Rows 9–11). When mSalvFont1.0’s unallocated scaffolds (assembled but unplaced sequences) were queried alongside chromosome-scale scaffolds, this exact high-quality pattern was replicated within the 34^th^ unplaced scaffold (scaffold_34_MOD), where it was represented eight times (Table 3, Rows 13–15; full data available <https://doi.org/10.5281/zenodo.21083596>). Therefore, scaffold_34_MOD putatively contains the Brook Trout master sex-determining gene. While further research is needed to validate this unplaced sequence as truly sex-linked, it would nonetheless be advantageous for future studies to include the 34^th^ unallocated scaffold with the 42 chromosome-scaffolds during variant discovery.

### Salmonid Comparisons

mSalvFont1.0 was benchmarked against North American Trout assemblies published in 2025 by aggregating metrics of accuracy and contiguity (Table 4). Overall, the Mid-Atlantic Brook Trout genome was larger, contained more complete BUSCOs, and still consisted of fewer contigs and scaffolds (mSalvFont1.0; 2.9 Gb; 99.3%; 3,333; 1,299), when compared to the Northern Brook Trout (ASM2944872v1; 2.5 Gb; 98.6%; 9,083; 2,721), Lahontan Cutthroat Trout (2.5 Gb; 98.0%; 3,738; 1,881), and Westslope Cutthroat Trout (2.4 Gb; 98.2%; 4,647; 3,266) assemblies. Furthermore, mSalvFont1.0 reported the second fewest gaps, tied for the highest QV score, and had the second highest k-mer completeness among the four assemblies (Table 4). These indices collectively indicated higher or equivalent accuracy and contiguity for the Mid-Atlantic Brook Trout assembly compared to other recently published North American Trout genomes.

Syntenic regions shared among Mid-Atlantic and Northern Brook Trout assemblies was scrutinized with a ribbon plot (Figure 3A), dot plot (Figure S1), and by comparing chromatin contact maps (Figure 3B). Both assemblies were subset to only include their 42 largest chromosome-scaffolds to reduce observational noise and aid visual juxtaposition. Genome-wide chromatin contact frequencies were consistently higher across the Mid-Atlantic Omni-C map than the Northern Hi-C map, suggesting a greater quantity of informative chromatin links were used during assembly of mSalvFont1.0. Differences in chromatin conformation resolution were likely driven by variation among the library preparation kits used. Dovetail Omni-C chemistry was used for mSalvFont1.0, an approach expected to yield greater and more even coverage of chromatin interactions than the restriction enzyme kit used in the assembly of ASM2944872v1 (Ramani et al. 2016). Examination of Brook Trout contact maps revealed a higher information content (denser and more widespread chromatin interactions) in the Mid-Atlantic assembly (Figure 3B), which informed the ordering and orientation of sequences during both semi-automated steps (e.g., contig and scaffold assembly) and manual curation.

### Inversion Validation

General synteny across the 42 assembled chromosomes was revealed following inspection of macrosyntenic blocks between Mid-Atlantic (mSalvFont1.0) and Northern (ASM2944872v1) Brook Trout genomes (Figure 3A). However, many segments appeared inverted among assemblies. Given that the Northern Brook Trout genome did not undergo manual curation, it is unclear whether these rearrangements reflect true structural variation or simply orientation errors introduced during the assembly process. mSalvFont1.0 contained fewer than half as many scaffolds compared to ASM2944872v1 (1,299 vs 2,721), nearly three times fewer contigs (3,333 vs 9,083), fewer than half as many gaps (2,034 vs 4,299), and slightly more complete BUSCOs (99.3% vs 98.6%), suggesting that the combined use of HiFi, UL, and Omni-C data produced a more complete genome assembly than the integration of HiFi, Hi-C, linkage mapping, and short-read polishing approach utilized by Lecomte et al. 2025.

As a single informative example, visualization of syntenic relationships within the eighth chromosome revealed an initially compelling inversion signal among Brook Trout lineages (Figure 4A). While numerous other chromosomes also displayed regions differing in orientation between assemblies, the lone inverted sequence visible in the center of the eighth chromosome offered a straightforward example of a potential structural variant given the general congruence of the dot plot outside this outlier locus. To validate this potential rearrangement, all combinations of chromatin conformation data and genome assemblies were used to produce four contact maps that were scrutinized narrowing in on the eighth chromosome, Sfo_8 and NC_074682.1, respectively (Figure 4B). A biologically valid inversion (i.e., not an assembly artefact) would be revealed during alignment of one lineage’s chromatin contact data to another’s genome assembly (Payne et al. 2025). The inversion signal does not appear when either the Northern Hi-C or Mid-Atlantic Omni-C data are aligned to the Mid-Atlantic genome but the signal is present when both sets of chromatin contact reads are aligned to the Northern genome, suggesting the inversion signal observed is diagnostic of either an assembly orientation error or a heterozygous variant. The latter seems biologically implausible (Figure 4B, bottom right panel), given that the specimen used to assemble the Northern Brook Trout genome was a double-haploid female and therefore should present entirely homozygous (i.e., not heterozygous for any inversions), further supporting that this signal is an assembly artefact and underscoring the value of manual curation. While this finding casts doubt on the fine-scale structural integrity of the Northern Brook Trout assembly, further inspection of inverted sequences between the lineages may uncover true structural variation.

### Reference Bias

Read mapping and variant identification statistics have been reported for both implementations of the snpArcher pipeline (Table 5), where otherwise identical iterations of the workflow only differed by lineage of chromosome-subset Brook Trout reference genome (Mid-Atlantic vs Northern). In theory, the short-read WGS dataset should align to mSalvFont1.0 without bias, as the reads and assembly itself were both generated from the same Mid-Atlantic Brook Trout specimen. Therefore, the statistics calculated from the VCF file using mSalvFont1.0 as the reference were considered to be the accurate and expected quantities for all comparative purposes. Percent *observed reference bias* (Table 5, column 4) was calculated as the difference between the Northern statistics (column 3) and Mid-Atlantic statistics (column 2), standardized by the Mid-Atlantic statistics (column 2). The number of reads mapped to the smaller, chromosome-subset Northern genome (2.17 Gb) was slightly greater than the number mapped to the larger chromosome-subset Mid-Atlantic genome (2.36 Gb) by 0.89% (Table 5). However, 10.8% more reads were flagged as duplicates and 0.32% fewer reads were properly paired when aligned to the Northern genome, indicative of mismapping. Overall, the average depth of coverage was 6.03% greater when the same reads were aligned to the Northern assembly when compared to the Mid-Atlantic genome, likely due to the Northern genome being 8.05% (0.19 Gb) smaller. In sum, these metrics indicate that the structurally divergent and smaller Northern genome (ASM2944872v1) drives increased misalignment of reads when mapping Mid-Atlantic Brook Trout sequences.

Raw variant calls identified 136.65% more SNPs, 131.87% more INDELs, 3,236.77% more multiallelic sites, and 26.94% more heterozygous sites when the same dataset was aligned to ASM2944872v1 as opposed to mSalvFont1.0. Following filtering, variant counts included 121.40% more SNPs, 114.93% more INDELs, 4,141.92% more multiallelic sites, and 20.74% more heterozygous sites in the ASM2944872v1-aligned dataset compared to the mSalvFont1.0-aligned dataset. While the greater counts of SNPs and INDELs observed using the Northern Brook Trout genome reflect fixed differences between the lineages (e.g., non-reference homozygotes), the vastly inflated numbers of multiallelic and heterozygous sites reveal a systematic failure to accurately map Mid-Atlantic reads to the Northern genome.

Considering the conservation relevance of genome-wide heterozygosity for informing population dynamics and management of imperiled lineages (Kardos et al. 2021; da Silva et al. 2025; McLaughlin et al. 2025; van Oosterhout et al. 2026), the presently described reference bias adds to mounting evidence that alignment to phylogenetically divergent assemblies can substantially inflate diversity metrics and misinform biological conclusions (Maurstad et al. 2025). Variant filtering tamped the magnitude of bias for all investigated polymorphism metrics except the number of multiallelic sites, which increased post-filtering. While filtering decreased heterozygosity bias by 6.2%, the persisting 20.74% inflation of this key metric remains highly problematic for conservation accuracy. Continued efforts to rigorously test how filtering parameters remedy reference bias may offer potential mitigation strategies (Rick et al. 2023).

## Conclusion

A *de novo*, chromosome-level genome for Mid-Atlantic Brook Trout was assembled by coalescing PacBio HiFi, ONT Ultra-Long, and Omni-C data with recently developed bioinformatic tools and manual curation software. mSalvFont1.0 is now the second available genome for the wide-ranging and conservation-relevant Brook Trout and expands phylogenetic representation to the Mid-Atlantic lineage. We anticipate that this assembly will help uncover genomic variation unique to divergent Brook Trout lineages, particularly in the more southern portion of the species’ distribution.

Quality and contiguity metrics indicate that mSalvFont1.0 improves upon recent North American Trout genomes, likely due to the inclusion of UL reads (i.e., N50 ≥ 50 Kb) and Omni-C-guided manual curation (Ghurye et al. 2019; Li & Durbin 2024). However, mSalvFont1.0 is not a gapless reference genome and has not been assessed for telomeric motifs or repetitive content within unallocated scaffolds. While overall completeness is high, the presence of 2,034 gaps and the inability to place 1,257 scaffolds within chromosomes preclude this assembly from achieving full continuity. Furthermore, this haploid genome lacks placement of the mobile and repetitive master sex-determining gene, sdY, within the 42 chromosome-scaffolds, thus complicating the identification of the Brook Trout sex chromosome.

The larger mSalvFont1.0 assembly (2.9 Gb) captures a snapshot of genomic variation unique to populations arising from the Mid-Atlantic glacial refugium, while the smaller Northern Brook Trout assembly (2.4 Gb; ASM2944872v1) represents variation likely stemming from the Acadian refugium. Substantial intraspecific variation in genome sizes has been identified within some plant species, where differences in DNA content among populations have been correlated with severity of environmental stress (i.e., populations with larger genomes appeared more drought tolerant) (Potapenko et al. 2026). Disentangling the eco-evolutionary impacts of genome size variation within Brook Trout lineages may illuminate how environmental gradients and repetitive genomic content interact to influence salmonid adaptation.

An assembly from the Mississippian refugial lineage (e.g., Georgia, USA) is needed to provide a more comprehensive representation of Brook Trout diversity across its range. While reduced-representation sequencing efforts have indicated Brook Trout from the southernmost end of their distribution (i.e., Mississippian refugium) harbor the greatest number of private alleles compared to other lineages (Meek et al. 2025), these estimates could be artificially inflated by reference bias (Maurstad et al. 2025; Mohn et al. 2025). Importantly, we establish that the Mid-Atlantic assembly (mSalvFont1.0) mitigates substantial reference bias that would otherwise occur if Mid-Atlantic-origin Brook Trout WGS reads were naively aligned to the Northern assembly (ASM2944872v1). Additionally, we highlight the nascent use of long-read and long-range sequencing technologies to further validate putative structural variation unique to divergent Brook Trout lineages that may be improperly characterized by short-reads alone (Nemeczek et al. 2025). Structurally accurate analyses of Brook Trout genomic variation will likely reveal enhanced insights into regional diversity and local adaptation that can be translated into conservation actions for this imperiled species (Stuart et al. 2026).

# Tables

**Table 1:** Reference genome metrics for Mid-Atlantic Brook Trout (mSalvFont1.0) calculated across major assembly phases. Note, the curated assembly is available on NCBI with the following link: <https://www.ncbi.nlm.nih.gov/datasets/genome/GCA_053542365.1/>.

| **Assembly Level** | **Scaffolded** | **Curated**  **(mSalvFont1.0)** | **Chromosome Subset**  **(42 chromosome-scaffolds)** |
| --- | --- | --- | --- |
| **Assembly Statistics** | | | |
| Total Length (Gb) | 3.00 | 2.91 | 2.36 |
| Chromosome Scale (%) | 0 | 81 | 100 |
| **Continuity Metrics** | | | |
| Number of Contigs | 3,369 | 3,333 | 1,623 |
| Number of Scaffolds | 1,391 | 1,299 | 42 |
| Contig N50 (Mb) | 3.3 | 3.3 | 3.9 |
| Scaffold N50 (Mb) | 68 | 55 | 58 |
| Gaps | 1,978 | 2,034 | 1,581 |
| **BUSCO Scores** | | | |
| Complete (%) | 98.8 | 99.3 | 94.7 |
| Duplicated (%) | 45.0 | 43.9 | 40.3 |
| Fragmented (%) | 0.9 | 0.2 | 0.1 |
| Missing (%) | 0.3 | 0.5 | 5.2 |

**Table 2:** Counts of mSalvFont1.0’s major annotated features using RNA-seq data from 11 Brook Trout tissue types (NCBI: PRJNA256998). Annotation and associated summary files are housed at <https://doi.org/10.5281/zenodo.21083596>.

| **Genes** | 43,790 |
| --- | --- |
| **mRNAs** | 69,297 |
| **Non-Coding RNAs** | 3,302 |
| **Pseudo Transcripts** | 5,281 |

**Table 3:** Alignment statistics for an mRNA partial coding sequence (CDS) from the Brook Trout master sex-determining gene, sdY. This 515-base-pair CDS was identified by Yanno et al. (2013) and is publicly available (NCBI Accession: JF826021). We queried the sdY CDS against the Mid-Atlantic (mSalvFont1.0) and Northern (ASM2944872v1) Brook Trout assemblies. Within chromosome-level scaffolds (Rows 1-7), we found three partial hits with high statistical support, suggesting that sdY is absent from the 42 chromosome-scaffolds, but that autosomal pseudogene copies are present in each assembly. However, on the 34^th^ unplaced scaffold of mSalvFont1.0 (scaffold_34_MOD), three hits with greater statistical support indicate a probable location for sdY (Rows 13-15).

| **Row Number** | **Assembly** | **Subject** | **Alignment Length (bp)** | **Identity (%)** | **E-Value** |
| --- | --- | --- | --- | --- | --- |
| 1 | mSalvFont1.0 | Sfo_22 | 299 | 71.2 | 1e-28 |
| 2 | mSalvFont1.0 | Sfo_6 | 233 | 69.9 | 5e-20 |
| 3 | mSalvFont1.0 | Sfo_31 | 392 | 65.1 | 5e-14 |
| 4 |  | | | | |
| 5 | ASM2944872v1 | NC_074695.1 | 299 | 71.2 | 1e-28 |
| 6 | ASM2944872v1 | NC_074671.1 | 233 | 69.9 | 5e-20 |
| 7 | ASM2944872v1 | NC_074689.1 | 392 | 65.1 | 5e-14 |
| 8 |  | | | | |
| 9 | Unassembled | HiFi Read 235997499 | 369 | 99.7 | 0.0 |
| 10 | Unassembled | HiFi Read 235997499 | 102 | 100 | 1e-42 |
| 11 | Unassembled | HiFi Read 235997499 | 46 | 100 | 3e-12 |
| 12 |  |  |  |  |  |
| 13 | mSalvFont1.0 | scaffold_34_MOD | 368 | 99.7 | 0.0 |
| 14 | mSalvFont1.0 | scaffold_34_MOD | 102 | 100 | 2e-44 |
| 15 | mSalvFont1.0 | scaffold_34_MOD | 46 | 100 | 6e-14 |

**Table 4:** Comparison of genome assembly metrics from endemic North American Trout assemblies published in 2025. Counts of contigs, scaffolds, and gaps enable comparison of genome contiguity, while identification of complete BUSCOs, quality value, and k-mer completeness reflect overall accuracy and completeness.

| **Assembly** | **Mid-Atlantic Brook Trout** | **Northern Brook Trout** | **Lahontan Cutthroat Trout** | **Westslope Cutthroat Trout** |
| --- | --- | --- | --- | --- |
| **Total Length (Gb)** | 2.9 | 2.5 | 2.5 | 2.4 |
| **Contigs** | 3,333 | 9,083 | 3,738 | 4,647 |
| **Scaffolds** | 1,299 | 2,721 | 1,881 | 3,266 |
| **Chromosomes** | 42 | 42 | 32 | 33 |
| **Gaps** | 2,034 | 4,299 | 1,857 | *N/A* |
| **Complete BUSCOs (%)** | 99.3 | 98.6 | 98.0 | 98.2 |
| **Duplicated BUSCOs (%)** | 43.9 | 41.7 | 42.0 | 43.1 |
| **Quality Value** | 59 | *N/A* | 59 | 43 |
| **K-mer Completeness** | 95 | *N/A* | 97 | 97 |
| **Publication** | This Study | Lecomte et al. 2025 | Payne et al. 2025 | Flores et al. 2025 |
| **NCBI Accession** | GCA_053542365.1 | GCA_029448725.1 | GCA_043774065.1 | GCA_045791955.2 |

**Table 5:** Read alignment and variant identification metrics for a Mid-Atlantic Brook Trout WGS sample mapped to both the Mid-Atlantic (mSalvFont1.0) and Northern (ASM2944872v1) genome assemblies. Bias was calculated as the difference between the Northern statistics (column 3) and Mid-Atlantic statistics (column 2), standardized by the Mid-Atlantic statistics (column 2). Variant statistics were calculated before and after filtering.

| **Brook Trout Reference Genome** | **Mid-Atlantic** | **Northern** | **Bias (%)** |
| --- | --- | --- | --- |
| **Alignment Statistics** | | | |
| Mapped Reads (%) | 97.10 | 97.96 | 0.89 |
| Duplicated Reads (%) | 13.61 | 15.08 | 10.80 |
| Properly Paired Reads (%) | 93.42 | 93.12 | -0.32 |
| Mean Read Depth | 36.67 | 38.88 | 6.03 |
| **Raw Variant Statistics** | | | |
| Raw SNPs | 3,272,011 | 7,743,291 | 136.65 |
| Raw INDELs | 811,863 | 1,882,491 | 131.87 |
| Raw Multiallelic Sites | 4,022 | 134,205 | 3,236.77 |
| Raw Heterozygous Sites | 3,257,754 | 4,135,231 | 26.94 |
| **Filtered Variant Statistics** | | | |
| Filtered SNPs | 3,156,503 | 6,988,462 | 121.40 |
| Filtered INDELs | 746,352 | 1,604,160 | 114.93 |
| Filtered Multiallelic Sites | 2,724 | 115,550 | 4,141.92 |
| Filtered Heterozygous Sites | 3,153,536 | 3,807,539 | 20.74 |

# Figures

**Figure 1:** **A)** Brook Trout sampling sites for the Mid-Atlantic genome assembly (mSalvFont1.0 – Dry Run, Virginia, USA, this study) and the Northern genome assembly (ASM2944872v1 – Cape Race, Newfoundland, CAN, Lecomte et al. 2025). **B)** A Mid-Atlantic Brook Trout sampled from Virginia, USA in 2023.

**Figure 2:** Assembly schematic outlining the construction of the Mid-Atlantic Brook Trout genome, mSalvFont1.0. Several auxiliary steps are not displayed to aid visual clarity but are documented in the methods. To highlight an important quality control measure that assesses attributes of the underlying HiFi data, the GenomeScope k-mer profile was displayed in the bottom left corner.

**Figure 3**: **A)** Synteny ribbon plot comparing the Mid-Atlantic (mSalvFont1.0) and Northern (ASM2944872v1) Brook Trout genomes reveals general sequence congruence, with possible chromosomal inversions or structural errors evident across assemblies. **B)** Chromosome-subset chromatin interaction maps generated with the four combinations of Brook Trout assemblies and Hi-C data from the Mid-Atlantic and Northern genomes, with a common contact frequency scale for heuristic purposes. The top left panel displays mSalvFont1.0, while the bottom right shows ASM2944872v1.

**Figure 4**: Cross-validation assessment of a putative structural variant between Mid-Atlantic and Northern Brook Trout lineages. **A)** Visualization of macrosyntenic blocks found in the eighth chromosome between Mid-Atlantic (mSalvFont1.0) and Northern (ASM2944872v1) Brook Trout genomes exposes a rearranged sequence. **B)** A finely textured view of the eighth chromosome from each contact map reveals a misassembled region in the Northern Brook Trout genome.

# Data availability

Data produced from this study can be retrieved through NCBI BioProject PRJNA1336308.

Raw HiFi, Omni-C, ONT, and Illumina sequences are available under NCBI BioSample SAMN52064996. The NCBI accession is GCA_053542365.1, and the NCBI master record is JBSFAW000000000. We archived AGP, annotation, repeat masked, VCF, pretext, and sdY BLASTN files on Zenodo at [https://doi.org/10.5281/zenodo.18805821](https://doi.org/10.5281/zenodo.21083596) and deposited all bioinformatic scripts <https://doi.org/10.5281/zenodo.21224411>.

# Acknowledgements

We thank Sally Painter and the Montana Fish, Wildlife and Parks Conservation Genomics Lab for assistance in preparing for specimen acquisition and transportation. We thank Kris Mueller for generating the map used in Figure 1A. We thank Zak Robinson for his robust knowledge of the system and for his input on the specimen collection. We thank Brad Fink and the Virginia Department of Wildlife Resources for assisting with field specimen collection. We thank Marcela Uliano-Silva, João Ferreira, Camilla Santos, Michael Paulini, and the Physalia team for their valuable courses “Eukaryotic Genome Assembly Using PacBio and Hi-C” and “Manual Genome Curation Using PretextView”.

# Author contributions

Samuel W. Rosenbaum (Conceptualization, Data curation, Formal analysis, Investigation, Methodology, Project administration, Resources, Software, Supervision, Validation, Visualization, Writing – original draft, Writing – review & editing)

Merly Escalona (Formal analysis, Investigation, Methodology, Software, Supervision, Validation, Visualization, Writing – review & editing)

Samuel A. May (Supervision, Validation, Writing – review & editing)

Andrew R. Whiteley (Conceptualization, Funding Acquisition, Project administration, Resources, Supervision, Validation, Writing – review & editing)

# Funding

This work was supported by the National Science Foundation (NSF) CAREER grant (DEB-1652278). This research was also supported by University of Montana Genomics Core and Montana INBRE Data Science Core, which are funded by the National Institute of General Medical Sciences (P20GM103474), the Office of the Vice President for Research and Creative Scholarship at the University of Montana, and the M. J. Murdock Charitable Trust. The content is solely the responsibility of the authors and does not necessarily represent the official views of the UMGC or the National Institutes of Health.

# Conflicts of interest

The author(s) declare no conflict of interest.

# Literature cited

Akopyan, M., Genchev, M., Armstrong, E. E., & Mooney, J. A. (2025). Reference genome choice compromises population genetic analyses. Cell, 188(24), 6939-6952.

Ali, O. A., O’Rourke, S. M., Amish, S. J., Meek, M. H., Luikart, G., Jeffres, C., & Miller, M. R. (2016). RAD capture (Rapture): flexible and efficient sequence-based genotyping. Genetics, 202(2), 389-400.

Ali, A., Waldbieser, G. C., Youngblood, R. C., Wheeler, P. A., Scheffler, B. E., Willis, S., ... & Palti, Y. (2026). De novo Genome Assemblies of Four Rainbow Trout Genetic Lines Reveal Structural Variants In Pursuit of a Pangenome Reference. Molecular Ecology Resources, 26(5), e70167.

Allendorf, F. W., Bassham, S., Cresko, W. A., Limborg, M. T., Seeb, L. W., & Seeb, J. E. (2015). Effects of crossovers between homeologs on inheritance and population genomics in polyploid-derived salmonid fishes. Journal of Heredity, 106(3), 217-227.

Altschul, S. F., Madden, T. L., Schäffer, A. A., Zhang, J., Zhang, Z., Miller, W., & Lipman, D. J. (1997). Gapped BLAST and PSI-BLAST: a new generation of protein database search programs. Nucleic Acids Research, 25(17), 3389-3402.

Ayllon, F., Solberg, M. F., Besnier, F., Fjelldal, P. G., Hansen, T. J., Wargelius, A., ... & Glover, K. A. (2020). Autosomal sdY pseudogenes explain discordances between phenotypic sex and DNA marker for sex identification in Atlantic salmon. Frontiers in Genetics, 11, 544207.

Astashyn, A., Tvedte, E. S., Sweeney, D., Sapojnikov, V., Bouk, N., Joukov, V., ... & Murphy, T. D. (2024). Rapid and sensitive detection of genome contamination at scale with FCS-GX. Genome Biology, 25(1), 60.

Beemelmanns, A., Bouchard, R., Michaelides, S., Normandeau, E., Jeon, H. B., Chamlian, B., ... & Moore, J. S. (2025). Development of SNP Panels from low‐coverage whole genome sequencing (lcWGS) to support indigenous fisheries for three salmonid species in northern Canada. Molecular Ecology Resources, 25(3), e14040.

Bertho, S., Herpin, A., Jouanno, E., Yano, A., Bobe, J., Parrinello, H., ... & Guiguen, Y. (2022). A nonfunctional copy of the salmonid sex-determining gene (sdY) is responsible for the “apparent” XY females in Chinook salmon, *Oncorhynchus tshawytscha*. G3, 12(2), jkab451.

Bertolotti, A. C., Layer, R. M., Gundappa, M. K., Gallagher, M. D., Pehlivanoglu, E., Nome, T., ... & Macqueen, D. J. (2020). The structural variation landscape in 492 Atlantic salmon genomes. Nature Communications, 11(1), 5176.

Bohling, J. (2020). Evaluating the effect of reference genome divergence on the analysis of empirical RADseq datasets. Ecology and Evolution, 10(14), 7585-7601.

Brandies, P., Peel, E., Hogg, C. J., & Belov, K. (2019). The value of reference genomes in the conservation of threatened species. Genes, 10(11), 846.

Brookes, B., Jeon, H. B., Derry, A. M., Post, J. R., Rogers, S. M., Humphries, S., & Fraser, D. J. (2022). Neutral and adaptive drivers of genomic change in introduced brook trout (*Salvelinus fontinalis*) populations revealed by pooled sequencing. Ecology and Evolution, 12(2), e8584.

Cisneros, A. F., Dibyachintan, S., Bédard, F., Aubé, S., Lemieux, P., & Landry, C. R. (2026). Evolutionary causes and consequences of gene duplication. Nature Reviews Genetics, 1-18.

Cheng, H., Asri, M., Lucas, J., Koren, S., & Li, H. (2024). Scalable telomere-to-telomere assembly for diploid and polyploid genomes with double graph. Nature Methods, 21(6), 967-970.

Christensen, K. A., Flores, A. M., Joshi, J., Shibata, K., Fujimoto, T., Koop, B. F., & Devlin, R. H. (2025). Masu salmon species complex relationships and sex chromosomes revealed from analyses of the masu salmon (*Oncorhynchus masou masou*) genome assembly. G3: Genes, Genomes, Genetics, 15(2), jkae278.

Coombe, L., Kazemi, P., Wong, J., Birol, I., & Warren, R. L. (2025). ntSynt: multi-genome synteny detection using minimizer graph mappings. BMC biology, 23(1), 367.

Coombe, L., Warren, R. L., & Birol, I. (2025). ntSynt-viz: visualizing synteny patterns across multiple genomes. bioRxiv, 2025-01.

da Silva, J. M., Bertola, L. D., DeWoody, J. A., Steeves, T., Sunnucks, P., Vilaça, S. T., & Hoban, S. (2025). Conserving Genetic and Genomic Diversity in Accordance with the Global Biodiversity Framework. Annual Review of Animal Biosciences, 14.

Danecek, P., Bonfield, J. K., Liddle, J., Marshall, J., Ohan, V., Pollard, M. O., ... & Li, H. (2021). Twelve years of SAMtools and BCFtools. Gigascience, 10(2), giab008.

Danzmann, R. G., Morgan II, R. P., Jones, M. W., Bernatchez, L., & Ihssen, P. E. (1998). A major sextet of mitochondrial DNA phylogenetic assemblages extant in eastern North American brook trout (*Salvelinus fontinalis*): distribution and postglacial dispersal patterns. Canadian Journal of Zoology, 76(7), 1300-1318.

De Coster, W., D’hert, S., Schultz, D. T., Cruts, M., & Van Broeckhoven, C. (2018). NanoPack: visualizing and processing long-read sequencing data. Bioinformatics, 34(15), 2666-2669.

Erdman, B., Larson, W., Mitro, M. G., Griffin, J. D., Rowe, D., Haglund, J., ... & Kinnison, M. T. (2024). Complications of estimating hatchery introgression in the face of rapid divergence: A case study in Brook Trout (*Salvelinus fontinalis*). Evolutionary Applications, 17(12), e70026.

Faber-Hammond, J. J., Phillips, R. B., & Brown, K. H. (2015). Comparative analysis of the shared sex-determination region (SDR) among salmonid fishes. Genome Biology and Evolution, 7(7), 1972-1987.

Flores, A. M., Christensen, K. A., Godin, T., Palti, Y., Campbell, M. R., Waldbieser, G. C., ... & Koop, B. F. (2025). The genome assembly of the westslope cutthroat Trout, *Oncorhynchus lewisi*, reveals interspecific chromosomal rearrangements with the rainbow Trout, *Oncorhynchus mykiss*. G3: Genes, Genomes, Genetics, 15(5), jkaf064.

Flynn, J. M., Hubley, R., Goubert, C., Rosen, J., Clark, A. G., Feschotte, C., & Smit, A. F. (2020). RepeatModeler2 for automated genomic discovery of transposable element families. Proceedings of the National Academy of Sciences, 117(17), 9451-9457.

Formenti, G., Abueg, L., Brajuka, A., Brajuka, N., Gallardo-Alba, C., Giani, A., ... & Jarvis, E. D. (2022). Gfastats: conversion, evaluation and manipulation of genome sequences using assembly graphs. Bioinformatics, 38(17), 4214-4216.

Formenti, G., Theissinger, K., Fernandes, C., Bista, I., Bombarely, A., Bleidorn, C., ... & Zammit, G. (2022). The era of reference genomes in conservation genomics. Trends in Ecology & Evolution, 37(3), 197-202.

Funk, W. C., McKay, J. K., Hohenlohe, P. A., & Allendorf, F. W. (2012). Harnessing genomics for delineating conservation units. Trends in Ecology & Evolution, 27(9), 489-496.

Ghurye, J., Rhie, A., Walenz, B. P., Schmitt, A., Selvaraj, S., Pop, M., ... & Koren, S. (2019). Integrating Hi-C links with assembly graphs for chromosome-scale assembly. PLoS Computational Biology, 15(8), e1007273.

Hale, M. C., McKinney, G. J., Bell, C. L., & Nichols, K. M. (2017). Using linkage maps as a tool to determine patterns of chromosome synteny in the genus Salvelinus. G3: Genes, Genomes, Genetics, 7(11), 3821-3830.

Howe, K., Chow, W., Collins, J., Pelan, S., Pointon, D. L., Sims, Y., ... & Wood, J. (2021). Significantly improving the quality of genome assemblies through curation. GigaScience, 10(1), giaa153.

Hudgell, M. A. B., Osborne, M. J., Salinas, I., & Turner, T. F. (2025). A Chromosome-Level Genome for Gila Trout (*Oncorhynchus gilae*) Provides a Novel Resource for Conservation and Management.

Hudy, M., Thieling, T. M., Gillespie, N., & Smith, E. P. (2008). Distribution, status, and land use characteristics of subwatersheds within the native range of Brook Trout in the eastern United States. North American Journal of Fisheries Management, 28(4), 1069-1085.

Hutchings, J. A. (1996). Adaptive phenotypic plasticity in Brook Trout, *Salvelinus fontinalis*, life histories. Ecoscience, 3(1), 25-32.

Jeon, H. B., Yates, M., Gallagher, B. K., & Fraser, D. J. (2024). Life’s a ditch: demographic history and environmental factors shape fine-scale local adaptation within small populations of Brook Trout. Canadian Journal of Fisheries and Aquatic Sciences, 82, 1-13.

Jones, M. W., Clay, D., & Danzmann, R. G. (1996). Conservation genetics of Brook Trout (*Salvelinus fontinalis*): population structuring in Fundy National Park, New Brunswick, and eastern Canada. Canadian Journal of Fisheries and Aquatic Sciences, 53(12), 2776-2791.

Kardos, M., Armstrong, E. E., Fitzpatrick, S. W., Hauser, S., Hedrick, P. W., Miller, J. M., ... & Funk, W. C. (2021). The crucial role of genome-wide genetic variation in conservation. Proceedings of the National Academy of Sciences, 118(48), e2104642118.

Kazyak, D. C., Lubinski, B. A., Kulp, M. A., Pregler, K. C., Whiteley, A. R., Hallerman, E., ... & King, T. L. (2022). Population genetics of Brook Trout in the southern Appalachian Mountains. Transactions of the American Fisheries Society, 151(2), 127-149.

Lecomte, L., Árnyasi, M., Ferchaud, A. L., Kent, M., Lien, S., Stenløkk, K., ... & Mérot, C. (2024). Investigating structural variant, indel and single nucleotide polymorphism differentiation between locally adapted Atlantic salmon populations. Evolutionary Applications, 17(3), e13653.

Lecomte, L., Ferchaud, A. L., Normandeau, E., Mérot, C., Langlois-Parisé, I., Therrien, J. C., ... & Fraser, D. (2025). Chromosome-level genome assembly of a doubled haploid Brook Trout (*Salvelinus fontinalis*). G3: Genes, Genomes, Genetics, 15(6), jkaf066.

Lee, G. M., & Wright Jr, J. E. (1981). Mitotic and meiotic analyses of Brook Trout, *Salvelinus fontinalis*. Journal of Heredity, 72(5), 321-327.

Li, H., Handsaker, B., Wysoker, A., Fennell, T., Ruan, J., Homer, N., ... & 1000 Genome Project Data Processing Subgroup. (2009). The sequence alignment/map format and SAMtools. Bioinformatics, 25(16), 2078-2079.

Li, H. (2013). Aligning sequence reads, clone sequences and assembly contigs with BWA-MEM. arXiv preprint arXiv:1303.3997.

Li, H., & Durbin, R. (2024). Genome assembly in the telomere-to-telomere era. Nature Reviews Genetics, 25(9), 658-670.

Liu, S., Gao, G., Layer, R. M., Thorgaard, G. H., Wiens, G. D., Leeds, T. D., ... & Palti, Y. (2021). Identification of high-confidence structural variants in domesticated rainbow trout using whole-genome sequencing. Frontiers in Genetics, 12, 639355.

Lubieniecki, K. P., Lin, S., Cabana, E. I., Li, J., Lai, Y. Y., & Davidson, W. S. (2015). Genomic instability of the sex-determining locus in Atlantic salmon (Salmo salar). G3: Genes, Genomes, Genetics, 5(11), 2513-2522.

Mamoozadeh, N. R., Whiteley, A. R., Letcher, B. H., Kazyak, D. C., Tarsa, C., & Meek, M. H. (2025). A new genomic resource to enable standardized surveys of SNPs across the native range of Brook Trout (*Salvelinus fontinalis*). Molecular Ecology Resources, 25(5), e13853.

Mamoozadeh, N., Cooper, A., Quinlan, H., Varian, A., Infante, D., & Meek, M. (2026). Dams and Introduced Species Drive Patterns of Environmental Adaptation in an Iconic but Imperiled Coldwater Fish (Brook Trout, Salvelinus fontinalis). Evolutionary Applications, 19(5), e70239.

Manni, M., Berkeley, M. R., Seppey, M., & Zdobnov, E. M. (2021). BUSCO: assessing genomic data quality and beyond. Current Protocols, 1(12), e323.

Marçais, G., Delcher, A. L., Phillippy, A. M., Coston, R., Salzberg, S. L., & Zimin, A. (2018). MUMmer4: A fast and versatile genome alignment system. PLoS Computational Biology, 14(1), e1005944.

Maurstad, M. F., Hoff, S. N. K., Cerca, J., Ravinet, M., Bradbury, I., Jakobsen, K. S., ... & Jentoft, S. (2025). Reference genome bias in light of species-specific chromosomal reorganization and translocations. Genome Biology, 26(1), 1-26.

May, S. A., Rosenbaum, S. W., Pearse, D. E., Kardos, M., Primmer, C. R., Baetscher, D. S., & Waples, R. S. (2025). The genomics revolution in nonmodel Species: predictions vs. reality for salmonids. Molecular Ecology, e17758.

McGinnis, S., & Madden, T. L. (2004). BLAST: at the core of a powerful and diverse set of sequence analysis tools. Nucleic Acids Research, 32(suppl_2), W20-W25.

McKenna, A., Hanna, M., Banks, E., Sivachenko, A., Cibulskis, K., Kernytsky, A., ... & DePristo, M. A. (2010). The Genome Analysis Toolkit: a MapReduce framework for analyzing next-generation DNA sequencing data. Genome research, 20(9), 1297.

McKinney, G. J., Nichols, K. M., & Ford, M. J. (2021). A mobile sex‐determining region, male‐specific haplotypes and rearing environment influence age at maturity in Chinook salmon. Molecular Ecology, 30(1), 131-147.

McLaughlin, C. M., Hinshaw, C., Sandoval-Arango, S., Zavala-Paez, M., & Hamilton, J. A. (2025). Redlisting genetics: towards inclusion of genetic data in IUCN Red List assessments. Conservation Genetics, 26(2), 213-223.

Meek, M. H., Mamoozadeh, N. R., Glaubitz, J. C., Hare, M. P., & Kraft, C. E. (2025). Range-wide climate risk and adaptive potential in a cold-water fish species. Nature Communications, 16(1), 7514.

Mérot, C., Stenløkk, K. S., Venney, C., Laporte, M., Moser, M., Normandeau, E., ... & Bernatchez, L. (2023). Genome assembly, structural variants, and genetic differentiation between lake whitefish young species pairs (*Coregonus* sp.) with long and short reads. Molecular Ecology, 32(6), 1458-1477.

Merriam, E. R., Petty, J. T., & Clingerman, J. (2019). Conservation planning at the intersection of landscape and climate change: brook trout in the Chesapeake Bay watershed. Ecosphere, 10(2), e02585.

Mirchandani, C. D., Shultz, A. J., Thomas, G. W., Smith, S. J., Baylis, M., Arnold, B., ... & Sackton, T. B. (2024). A fast, reproducible, high-throughput variant calling workflow for population genomics. Molecular Biology and Evolution, 41(1), msad270.

Mohn, R. A., Garner, M., Manos, P. S., & Hipp, A. L. (2026). Variant calling in the goldilocks zone: How reference genome choice and read mapping stringency impact heterozygosity estimates and phylogenetic analyses. Molecular Ecology Resources, 26(1), e70079.

Nemeczek, C. M., Delgado, M. L., Smith, M. E., MacMillan, J., Van Wyngaarden, M., & Ruzzante, D. E. (2025). The potential role of haploblocks in the persistence of small, isolated populations of Brook Trout (*Salvelinus fontinalis*). Ecology and Evolution, 15(9), e72075.

Open2C, Abdennur, N., Fudenberg, G., Flyamer, I. M., Galitsyna, A. A., Goloborodko, A., ... & Venev, S. V. (2024). Pairtools: from sequencing data to chromosome contacts. PLOS Computational Biology, 20(5), e1012164.

Paez, S., Kraus, R. H., Shapiro, B., Gilbert, M. T. P., Jarvis, E. D., Vertebrate Genomes Project Conservation Group, ... & Venkatesh, B. (2022). Reference genomes for conservation. Science, 377(6604), 364-366.

Pasquier, J., Cabau, C., Nguyen, T., Jouanno, E., Severac, D., Braasch, I., ... & Bobe, J. (2016). Gene evolution and gene expression after whole genome duplication in fish: the PhyloFish database. BMC genomics, 17(1), 368.

Payne, C. Y., Escalona, M., Kieran Blair, S. R., Finger, A. J., Green, R. E., Pearse, D., ... & Garza, J. C. (2026). A genome assembly for Lahontan Cutthroat Trout, *Oncorhynchus clarkii henshawi*. Journal of Heredity, 117(1), 159-172.

Pflug, J. M., Holmes, V. R., Burrus, C., Johnston, J. S., & Maddison, D. R. (2020). Measuring genome sizes using read-depth, k-mers, and flow cytometry: methodological comparisons in beetles (*Coleoptera*). G3: Genes, Genomes, Genetics, 10(9), 3047-3060.

Potapenko, E., Shermeister, B., Mandel, T., & Hübner, S. (2026). Intraspecific genome size variation is attributed to adaptive silencing of transposable elements in Hordeum species. Molecular Biology and Evolution, 43(3), msag051.

Prasad, A., Lorenzen, E. D., & Westbury, M. V. (2022). Evaluating the role of reference‐genome phylogenetic distance on evolutionary inference. Molecular Ecology Resources, 22(1), 45-55.

Ramani, V., Cusanovich, D. A., Hause, R. J., Ma, W., Qiu, R., Deng, X., ... & Duan, Z. (2016). Mapping 3D genome architecture through in situ DNase Hi-C. Nature Protocols, 11(11), 2104-2121.

Ranallo-Benavidez, T. R., Jaron, K. S., & Schatz, M. C. (2020). GenomeScope 2.0 and Smudgeplot for reference-free profiling of polyploid genomes. Nature Communications, 11(1), 1432.

Rick, J. A., Brock, C. D., Lewanski, A. L., Golcher-Benavides, J., & Wagner, C. E. (2024). Reference genome choice and filtering thresholds jointly influence phylogenomic analyses. Systematic Biology, 73(1), 76-101.

Rhie, A., Walenz, B. P., Koren, S., & Phillippy, A. M. (2020). Merqury: reference-free quality, completeness, and phasing assessment for genome assemblies. Genome Biology, 21(1), 245.

Rhie, A., McCarthy, S. A., Fedrigo, O., Damas, J., Formenti, G., Koren, S., ... & Jarvis, E. D. (2021). Towards complete and error-free genome assemblies of all vertebrate species. Nature, 592(7856), 737-746.

Robinson, Z. L., Coombs, J. A., Hudy, M., Nislow, K. H., Letcher, B. H., & Whiteley, A. R. (2017). Experimental test of genetic rescue in isolated populations of Brook Trout. Molecular Ecology, 26(17), 4418-4433.

Robinson, Z. L., Coombs, J. A., Hudy, M., Nislow, K. H., & Whiteley, A. R. (2024). Estimates of effective number of breeders identify drivers of decline in Mid‐Atlantic Brook Trout populations. Evolutionary Applications, 17(10), e13769.

Rondeau, E. B., Christensen, K. A., Johnson, H. A., Sakhrani, D., Biagi, C. A., Wetklo, M., ... & Devlin, R. H. (2023). Insights from a chum salmon (*Oncorhynchus keta*) genome assembly regarding whole-genome duplication and nucleotide variation influencing gene function. G3: Genes, Genomes, Genetics, 13(8), jkad127.

Schwinghamer, C. W., Hartman, K. J., & Welsh, A. B. (2024). Genetic predictors of population resilience: A case study of native Brook Trout in headwater streams. North American Journal of Fisheries Management, 44(3), 535-550.

Sim, S. B., Corpuz, R. L., Simmonds, T. J., & Geib, S. M. (2022). HiFiAdapterFilt, a memory efficient read processing pipeline, prevents occurrence of adapter sequence in PacBio HiFi reads and their negative impacts on genome assembly. BMC Genomics, 23(1), 157.

Simão, F. A., Waterhouse, R. M., Ioannidis, P., Kriventseva, E. V., & Zdobnov, E. M. (2015). BUSCO: assessing genome assembly and annotation completeness with single-copy orthologs. Bioinformatics, 31(19), 3210-3212.

Śliwińska-Jewsiewicka, A., Kuciński, M., Kirtiklis, L., Dobosz, S., Ocalewicz, K., & Jankun, M. (2015). Chromosomal characteristics and distribution of rDNA sequences in the Brook Trout *Salvelinus fontinalis* (Mitchill, 1814). Genetica, 143(4), 425-432.

Smit, A. F. A., Hubley, R., & Green, P. (2015). RepeatMasker Open-4.0. 2013–2015.

Stuart, K. C., Oomen, R. A., Tigano, A., Wellenreuther, M., Wold, J., Field, D. L., & Mérot, C. (2026). A beginner's guide to structural variants in eco‐evolutionary population genomics. Molecular Ecology, 35(2), e70216.

Tigano, A., & Russello, M. A. (2022). The genomic basis of reproductive and migratory behaviour in a polymorphic salmonid. Molecular Ecology, 31(24), 6588-6604.

Tigano, A., Weir, T., Ward, H. G., Gale, M. K., Wong, C. M., Eliason, E. J., ... & Russello, M. A. (2023). Genomic vulnerability of a freshwater salmonid under climate change. Evolutionary Applications, 17(2), e13602.

Thorburn, D. M. J., Sagonas, K., Binzer‐Panchal, M., Chain, F. J., Feulner, P. G., Bornberg‐Bauer, E., ... & Eizaguirre, C. (2023). Origin matters: Using a local reference genome improves measures in population genomics. Molecular Ecology Resources, 23(7), 1706-1723.

Uliano-Silva, M., Ferreira, J. G. R., Krasheninnikova, K., Formenti, G., Abueg, L., Torrance, J., ... & McCarthy, S. A. (2023). MitoHiFi: a python pipeline for mitochondrial genome assembly from PacBio high fidelity reads. BMC Bioinformatics, 24(1), 288.

van Oosterhout, C., Speak, S. A., Birley, T., Hitchings, L. W., Bortoluzzi, C., Percival-Alwyn, L., ... & Morales, H. E. (2026). Genomic erosion in the assessment of species’ extinction risk and recovery potential. Journal of Heredity, esag011.

Waples, R. S. (1991). Pacific salmon, *Oncorhynchus* spp., and the definition of" species" under the Endangered Species Act. Marine Fisheries Review, 53(3), 11-22.

Webster, N., Price, S., Gowan, C., & Coster, S. S. (2021). A nonlethal genetic assay for discriminating sex in Brook Trout. North American Journal of Fisheries Management, 41(4), 1120-1123.

White, S. L., Rash, J. M., & Kazyak, D. C. (2023). Is now the time? Review of genetic rescue as a conservation tool for Brook Trout. Ecology and Evolution, 13(5), e10142.

Whiteley, A. R., Penaluna, B. E., Taylor, E. B., Weiss, S., Abadia-Cardoso, A., Gomez-Uchida, D., ... & Trotter, P. (2019). Trout and char: taxonomy, systematics, and phylogeography. Trout and Char of the World, 95-140.

Wood, D. E., Lu, J., & Langmead, B. (2019). Improved metagenomic analysis with Kraken 2. Genome Biology, 20(1), 257.

Yano, A., Nicol, B., Jouanno, E., Quillet, E., Fostier, A., Guyomard, R., & Guiguen, Y. (2013). The sexually dimorphic on the Y‐chromosome gene (sdY) is a conserved male‐specific Y‐chromosome sequence in many salmonids. Evolutionary Applications, 6(3), 486-496.

Zhou, C., McCarthy, S. A., & Durbin, R. (2023). YaHS: yet another Hi-C scaffolding tool. Bioinformatics, 39(1), btac808.

# Supplementary

## Tables

**Table S1:** Bioinformatic programs used during assembly.

| **Purpose** | **Software** | **Version** | **Non-Default Parameters** |
| --- | --- | --- | --- |
| Sequencing statistics | NanoStat | 1.6.0 | N/A |
| HiFi adapter removal | HiFiAdapterFilt | 3.0.0 | -l 44  -m 97 |
| ONT adapter removal | Dorado | 0.9.1 | --sequencing-kit SQK-LSK114  --emit-fastq |
| K-mer analysis | meryl | 1.4.1 | k=21 |
| Genome parameter estimation | GenomeScope2 | 2.0.1 | k=21  ploidy=2 |
| Contigging | hifiasm | 0.25.0 | --primary  --h1  --h2  --ul  --ul-rate 0.025  --l 3  --telo-m CCCTAA |
| File format conversion | gfatools | 0.5 | N/A |
| Assembly metrics | gfastats | 1.3.10 | --stats |
| Omni-C alignment | bwa mem | 0.7.19 | -5SP  -T0 |
| Pairs file generation | pairtools | 1.1.2 | --min-mapq 40  --walks-policy 5unique  --max-inter-align-gap 30  --mark-dups |
| BAM file generation | samtools | 1.21 | N/A |
| Hi-C map generation | PretextMap | 0.1.9 | --sortby length  --sortorder descend  --mapq 0 |
| View Hi-C map | PretextSnapshot | 0.0.5 | --sequences “full”  --resolution 4000  --colourMap 24 |
| Scaffolding | YaHS | 1.2.2 | --no-contig-ec  --no-scaffold-ec |
| Track generation for manual curation | Wellcome Sanger Institute’s rapid curation pipeline | 1.4.1 | N/A |
| Manual curation | PretextView | 1.0.3 | N/A |
| Gap closing | YAGCloser | 1.0.0 | -f 20  -mins 2  -mcc 2  -prt 0.25  -eft 0.2  -pld 0.2 |
| Mitochondrial genome assembly | MitoHiFi | 3.2.3 | -c mSalvFont1.0 contigs  -f NC_00860.1.fasta  -g NC_00860.1.gb |
| Genome-to-genome alignment | mummer4 | 4.0.1 | -r  -q  -c |
| Dot plot generation | dotPlotly | N/A | -x  -t  -l  -v |
| Gene completeness | busco | 6.0.0 | -l actinopterygii_obd10 |
| Quality value calculation | Merqury | 1.3 | N/A |
| Contamination screening | Kraken2 | 2.14 | --confidence 0.1  --use-names |
| Contamination screening | fcs-gx | 0.5.5 | --tax-id 8038 |
| Gene annotation | EGAPx | 0.5.2 | N/A |
| sdY presence/absence identification | BLASTN | 2.17.0 | -outfmt “7 std qcovs sstrand”  -evalue 1e-5 |
| Macroynteny block identification | ntSynt | 1.0.7 | --divergence 0.2 |
| Ribbon plot generation | ntSynt-viz | 1.0.7 | N/A |
| Repeat modeling | RepeatModeler | 2.0.8 | N/A |
| Repeat masking | RepeatMasker | 4.2.3 | N/A |
| Variant calling | snpArcher | 2.0.0-dev | expected_coverage: "high" |
| Variant statistics and filtering | BCFtools | 1.23.1 | --exclude 'QUAL < 30 \|\| FORMAT/DP < 10 \|\| FORMAT/DP > 100 \|\| FORMAT/GQ < 20'  --soft-filter "LowConf"  --mode + |

**Table S2:** Summary statistics of raw PacBio HiFi and ONT Simplex reads in base pairs (bp).

| **Metric** | **Raw HiFi** | **Trimmed HiFi** | **Raw ONT** | **Trimmed ONT** |
| --- | --- | --- | --- | --- |
| Mean read length (bp) | 18,514 | 18,514 | 50,077 | 50,052 |
| Mean read quality | 23 | 23 | 16 | 16 |
| Median read length (bp) | 17,845 | 17,845 | 43,812 | 43,789 |
| Median read quality | 26 | 26 | 19 | 19 |
| Number of reads | 8,481,671 | 8,481,627 | 322,630 | 322,630 |
| Read length N50 (bp) | 18,170 | 18,170 | 50,064 | 50,043 |
| Standard deviation of read length (bp) | 2,967 | 2,967 | 20,572 | 20,571 |
| Total bases | 157,030,510,034 | 157,029,659,646 | 16,156,175,272 | 16,148,232,778 |

**Table S3:** Omni-C statistics following alignment to contigs.

| Total Read Pairs | 422,880,978 | 100% |
| --- | --- | --- |
| Unmapped Read Pairs | 116,019,054 | 27.44% |
| Mapped Read Pairs | 136,650,220 | 32.31% |
| PCR Dup Read Pairs | 88,521,918 | 20.93% |
| No-Dup Read Pairs | 48,128,302 | 11.38% |
| No-Dup Cis Read Pairs | 20,048,727 | 41.66% |
| No-Dup Trans Read Pairs | 28,079,575 | 58.34% |
| No-Dup Valid Read Pairs (cis >= 1kb + trans) | 41,949,610 | 87.16% |
| No-Dub Cis Read Pairs < 1kb | 6,178,692 | 12.84% |
| No-Dup Cis Read Pairs >= 1kb | 13,870,035 | 28.82% |
| No-Dup Cis Read Pairs >= 10kb | 10,240,572 | 21.28% |

**Table S4:** Omni-C statistics following alignment to scaffolds.

| Total Read Pairs | 422,880,978 | 100% |
| --- | --- | --- |
| Unmapped Read Pairs | 113,844,178 | 26.92% |
| Mapped Read Pairs | 139,952,160 | 33.09% |
| PCR Dup Read Pairs | 88,870,200 | 21.02% |
| No-Dup Read Pairs | 51,081,960 | 12.08% |
| No-Dup Cis Read Pairs | 30,017,843 | 58.76% |
| No-Dup Trans Read Pairs | 21,064,117 | 41.24% |
| No-Dup Valid Read Pairs (cis >= 1kb + trans) | 44,903,290 | 87.9% |
| No-Dub Cis Read Pairs < 1kb | 6,178,670 | 12.1% |
| No-Dup Cis Read Pairs >= 1kb | 23,839,173 | 46.67% |
| No-Dup Cis Read Pairs >= 10kb | 20,206,115 | 39.56% |

**Table S5:** Characterization of repetitive content within the 42 chromosome-scale scaffolds in mSalvFont1.0.

| **Classification** | **Number of Elements** | **Length of Genome Occupied (bp)** | **% of Genome Occupied** |
| --- | --- | --- | --- |
| **Retroelements** | 765539 | 335203085 | 14.21 |
| **SINEs** | 32981 | 7691711 | 0.33 |
| **Penelope** | 2949 | 558870 | 0.02 |
| **LINEs** | 495006 | 224374495 | 9.51 |
| **CRE/SLACS** | 0 | 0 | 0 |
| **L2/CR1/Rex** | 353246 | 167942442 | 7.12 |
| **R1/LOA/Jockey** | 29198 | 5773922 | 0.24 |
| **R2/R4/NeSL** | 1002 | 730897 | 0.03 |
| **RTE/Bov-B** | 31275 | 16990116 | 0.72 |
| **L1/CIN4** | 13909 | 7101349 | 0.3 |
| **LTR elements** | 234603 | 102578009 | 4.35 |
| **BEL/Pao** | 1593 | 726020 | 0.03 |
| **Ty1/Copia** | 466 | 410206 | 0.02 |
| **Gypsy/DIRS1** | 198295 | 86839065 | 3.68 |
| **Retroviral** | 7001 | 5221622 | 0.22 |
| **DNA transposons** | 1188490 | 497291989 | 21.08 |
| **hobo-Activator** | 143530 | 42087835 | 1.78 |
| **Tc1-IS630-Pogo** | 569265 | 277692601 | 11.77 |
| **En-Spm** | 0 | 0 | 0 |
| **MULE-MuDR** | 0 | 0 | 0 |
| **PiggyBac** | 116570 | 97583249 | 4.14 |
| **Tourist/Harbinger** | 6453 | 1791109 | 0.08 |
| **Other (Mirage, P-element, Transib)** | 1453 | 152367 | 0.01 |
| **Rolling-circles** | 0 | 0 | 0 |
| **Unclassified** | 1364177 | 393959754 | 16.7 |
| **Total interspersed repeats** |  | 1226454828 | 51.99 |
| **Small RNA** | 71629 | 17392608 | 0.74 |
| **Satellites** | 0 | 0 | 0 |
| **Simple repeats** | 566732 | 51895981 | 2.2 |
| **Low complexity** | 71381 | 7217930 | 0.31 |

**Table S6:** Comprehensive counts of annotation features.

| **Type** | **Subtype** | **Counts** |
| --- | --- | --- |
| genes | other | 0 |
| genes | non-transcribed pseudo | 5108 |
| genes | transcribed pseudo | 0 |
| genes | has variants | 15023 |
| genes | partial | 128 |
| genes | Ig TCR segment | 22 |
| genes | non coding | 1847 |
| genes | protein coding | 36813 |
| genes | major correction | 706 |
| genes | minor correction | 0 |
| genes | premature stop | 160 |
| genes | has frameshifts | 628 |
| mRNAs | exon <= 3nt | 30 |
| mRNAs | partial | 128 |
| mRNAs | correction | 706 |
| mRNAs | model | 69297 |
| mRNAs | fully supported | 66587 |
| mRNAs | ab initio > 5% | 1238 |
| mRNAs | has gaps | 0 |
| mRNAs | model with correction | 706 |
| non-coding RNAs | exon <= 3nt | 0 |
| non-coding RNAs | partial | 0 |
| non-coding RNAs | correction | 1 |
| non-coding RNAs | model | 3302 |
| non-coding RNAs | fully supported | 3294 |
| non-coding RNAs | ab initio > 5% | 0 |
| non-coding RNAs | has gaps | 0 |
| pseudo transcripts | exon <= 3nt | 1 |
| pseudo transcripts | partial | 0 |
| pseudo transcripts | correction | 0 |
| pseudo transcripts | model | 5281 |
| pseudo transcripts | fully supported | 2581 |
| pseudo transcripts | ab initio > 5% | 0 |
| pseudo transcripts | has gaps | 0 |
| CDSs | exon <= 3nt | 1211 |
| CDSs | partial | 128 |
| CDSs | correction | 706 |
| CDSs | model | 69297 |
| CDSs | fully supported | 66587 |
| CDSs | ab initio > 5% | 1385 |
| CDSs | model with correction | 706 |
| CDSs | major correction | 706 |
| CDSs | minor correction | 0 |
| CDSs | premature stop | 160 |
| CDSs | has frameshifts | 628 |

## Figures

**Figure S1:** A synteny dot plot comparing the Mid-Atlantic (mSalvFont1.0) and Northern (ASM2944872v1) Brook Trout genomes.
